# Pulsatile arterial wall-blood flow interaction with wall pre-stress computed using an inverse algorithm

**DOI:** 10.1186/1475-925X-14-S1-S18

**Published:** 2015-01-09

**Authors:** Ashish Das, Anup Paul, Michael D Taylor, Rupak K Banerjee

**Affiliations:** 1 Department of Mechanical and Materials Engineering, University of Cincinnati, Cincinnati, OH 45221, USA; 2 Cincinnati Children's Hospital Medical Center, The Heart Institute, 3333 Burnet Avenue, Cincinnati, OH 45219, USA

## Abstract

**Background:**

The computation of arterial wall deformation and stresses under physiologic conditions requires a coupled compliant arterial wall-blood flow interaction model. The *in-vivo *arterial wall motion is constrained by tethering from the surrounding tissues. This tethering, together with the average *in-vivo *pressure, results in wall pre-stress. For an accurate simulation of the physiologic conditions, it is important to incorporate the wall pre-stress in the computational model. The computation of wall pre-stress is complex, as the un-loaded and un-tethered arterial shape with residual stress is unknown. In this study, the arterial wall deformation and stresses in a canine femoral artery under pulsatile pressure was computed after incorporating the wall pre-stresses. A nonlinear least square optimization based *inverse *algorithm was developed to compute the *in-vivo *wall pre-stress.

**Methods:**

First, the proposed *inverse *algorithm was used to obtain the un-loaded and un-tethered arterial geometry from the unstressed *in-vivo *geometry. Then, the un-loaded, and un-tethered arterial geometry was pre-stressed by applying a mean *in-vivo *pressure of 104.5 mmHg and an axial stretch of 48% from the un-tethered length. Finally, the physiologic pressure pulse was applied at the inlet and the outlet of the pre-stressed configuration to calculate the *in-vivo *deformation and stresses. The wall material properties were modeled with an incompressible, Mooney-Rivlin model derived from previously published experimental stress-strain data (Attinger et al., 1968).

**Results:**

The un-loaded and un-tethered artery geometry computed by the inverse algorithm had a length, inner diameter and thickness of 35.14 mm, 3.10 mm and 0.435 mm, respectively. The pre-stressed arterial wall geometry was obtained by applying the *in-vivo *axial-stretch and average *in-vivo *pressure to the un-loaded and un-tethered geometry. The length of the pre-stressed artery, 51.99 mm, was within 0.01 mm (0.019%) of the *in-vivo *length of 52.0 mm; the inner diameter of 3.603 mm was within 0.003 mm (0.08%) of the corresponding *in-vivo *diameter of 3.6 mm, and the thickness of 0.269 mm was within 0.0015 mm (0.55%) of the *in-vivo *thickness of 0.27 mm. Under physiologic pulsatile pressure applied to the pre-stressed artery, the time averaged longitudinal stress was found to be 42.5% higher than the circumferential stresses. The results of this study are similar to the results reported by Zhang et al., (2005) for the left anterior descending coronary artery.

**Conclusions:**

An inverse method was adopted to compute physiologic pre-stress in the arterial wall before conducting pulsatile hemodynamic calculations. The wall stresses were higher in magnitude in the longitudinal direction, under physiologic pressure after incorporating the effect of *in-vivo *axial stretch and pressure loading.

## Background

The computation of arterial wall deformation and stresses under physiologic conditions requires a mathematical model of coupled compliant arterial wall-blood flow interaction. The *in-vivo *arterial wall motion is constrained by tethering from the surrounding tissues. This tethering, together with the average *in-vivo *pressure, results in wall pre-stress. The release of this pre-stress in an excised artery results in its longitudinal and radial retraction [1-3]. The longitudinal stretch required to elongate the artery from the excised length to the *in-vivo *length is known as *in-vivo *axial stretch. The excised artery sample without the arterial pressure and longitudinal stretch is known as the un-tethered load-free artery. The pre-stressed arterial configuration is obtained by applying the *in-vivo *longitudinal stretch and mean arterial pressure to the un-tethered, load-free arterial configuration. Thereafter, the pulsatile pressure load is applied to the pre-stressed arterial geometry to compute the interaction between the blood-flow and arterial wall. For an accurate simulation of the physiologic conditions, it is important to incorporate the wall pre-stress. Excessive deformation will result, if the *in-vivo *pressure load is applied to the *in-vivo *arterial geometry without accounting for the wall pre-stress.

Longitudinal shrinkage of the order of 48% has been reported by Van Loom, [4], in a study of a canine femoral artery. Huang et al., [5], have reported a similar value of 33% for longitudinal shrinkage and 12% to 16% circumferential shrinkage for human carotid arteries with a plaque deposit. Considerable variation in the *in-vivo *axial stretch along the arterial vasculature has been reported by Guo et al., [6], Hamza et al., [7], and Algranti et al., [8], for both porcine aorta and coronary arteries. The level of axial pre-stress has also been reported to vary with age and disease condition [9,10]. Therefore, to accurately determine the state of stress and strain in an arterial branch under physiologic condition it is important to account for the *in-vivo *arterial wall pre-stress resulting from the *in-vivo *axial stretch and mean physiologic pressure.

The computation of wall pre-stress is complex, as the un-loaded and un-tethered arterial shape with residual stress is unknown. A manual trial-and-error based procedure to compute the load-free artery geometry has been developed by Huang et al., [11], for a carotid artery with plaque and Tang et al., [12], for a diseased coronary artery. A similar methodology has been adopted for an idealized axi-symmetric arterial geometry by Sinha-Roy et al., [13] for a canine femoral artery, and by Konala et al., [14] for a human coronary stenosis with anisotropic material model. However, manual trial-and-error process may lead to variability in the arterial shape and pre-stress values. Such process may not have unique solution.

The research in automatic inverse computation can be categorized into two classes: 1) the direct method of solving the inverse elastostatics boundary value problem, and 2) geometrical shape matching algorithms to match the pre-stressed arterial shape with the *in-vivo *shape. The direct method was adopted by Lu et al., [15], to compute pre-stressed arterial geometry of a patient-specific abdominal aortic aneurysm (AAA). The mathematical formulation for this methodology is complex [15,16], which makes it difficult to program the algorithm in an existing finite element formulation. The boundary conditions for the inverse boundary value problem have no physical significance [16] and the models with incompressible material have been reported to be prone to ill-conditioned numerical convergence [16]. Therefore, shape-matching algorithms have been more commonly adopted for such problems [17-21].

The shape matching algorithms iteratively modify the arterial geometry until a geometrical configuration similar to the *in-vivo *shape that is in stress-equilibrium with the applied *in-vivo *loads, is obtained [18,21-23]. Some of the shape matching algorithms are based on optimization of an objective function that measures the deviation of the computed shape from the *in-vivo *shape [18,22]. All shape matching algorithms iteratively update the nodal locations of the computational model. This essentially implies that the position of each arterial wall node is an optimization variable. For a patient-specific geometry with a large number of nodes, this can increase the number of optimization variables, causing the convergence process to be non-trivial.

Bolls et al., [21], have implemented an algorithm that iteratively modifies the *in-vivo *shape by subtracting the displacements resulting from the application of *in-vivo *pressure load. Putter et al., [20], and Gee et al., [23], have proposed similar algorithms that incrementally apply pressure load leading to full *in-vivo *pressure, while maintaining stress equilibrium by updating deformation gradients and strain tensors. All of the above mentioned algorithms have been mainly applied to AAA cases which have lower axial stretch. For a patient-specific AAA geometry, assuming that the direction of deformation to be predominantly radial, Raghavan et al., [18] and Lu et al., [22] simplify the optimization problem to a single variable. However, such assumptions are not valid for arterial wall under axial stretch. In most of the proposed algorithms, the stress equilibrium state is computed using only the mean arterial pressure. However, the importance of incorporating viscous flow induced stress has been reported by Hsu et al., [24] study.

The objectives of this research were: a) to develop and test an optimization based inverse algorithm to compute the load-free and pre-stressed arterial geometry from *in-vivo *data, and b) to investigate the effect of *in-vivo *axial stretch on the state of arterial stress, under a pulsatile pressure-flow condition for a physiological model of a canine femoral artery. A novel optimization-based inverse algorithm was implemented, that is applicable for any patient-specific arterial geometry. It can incorporate material non-linearity as well as large deformations and strains resulting from the *in-vivo *axial stretch. The main difference between the proposed algorithm and previously developed optimization based inverse algorithms [17,18,20,21,25] is in simplifying the optimization formulation into a two variable problem. This was achieved by developing a finite element model of arterial shrinking and the arterial expansion under *in-vivo *axial stretch and pressure. For any geometry, this simplification reduces the number of optimization variables to just two variables: the axial shrink and the radial shrink. Unlike other algorithms [17,18,20,21,25], the conservation of the volume of the *in-vivo *arterial wall was enforced by incorporating an optimization constraint. This ensured that the geometrical modifications of the arterial wall in optimization iterations maintained the *in-vivo *wall volume.

## Methods

The inverse method was specifically developed for any patient-specific arterial geometry. As a first step, an idealized arterial geometry with physiologic pressure load was used as a testcase in this study. The inverse method presented here mimics the exact steps for any patient-specific case to be tested in future. First, the pre-stressed arterial wall geometry was computed using the proposed inverse method. Then, the pulsatile pressure load was applied at the inlet and the outlet of the pre-stressed artery to compute the transient wall blood-flow interaction.

### Arterial geometry

A straight arterial segment of a canine femoral artery of length of 52 mm, inner radius of 1.8 mm and thickness of 0.27 mm was adopted for this study [13]. This geometry is a simplified version of the tapered femoral artery studied by Sinha-Roy et al, [13]. In this study, the taper due to the reduction of 0.1 mm radius from the inlet to the outlet over the 52 mm length (angle of 0.11°) was neglected.

**Figure 1:**
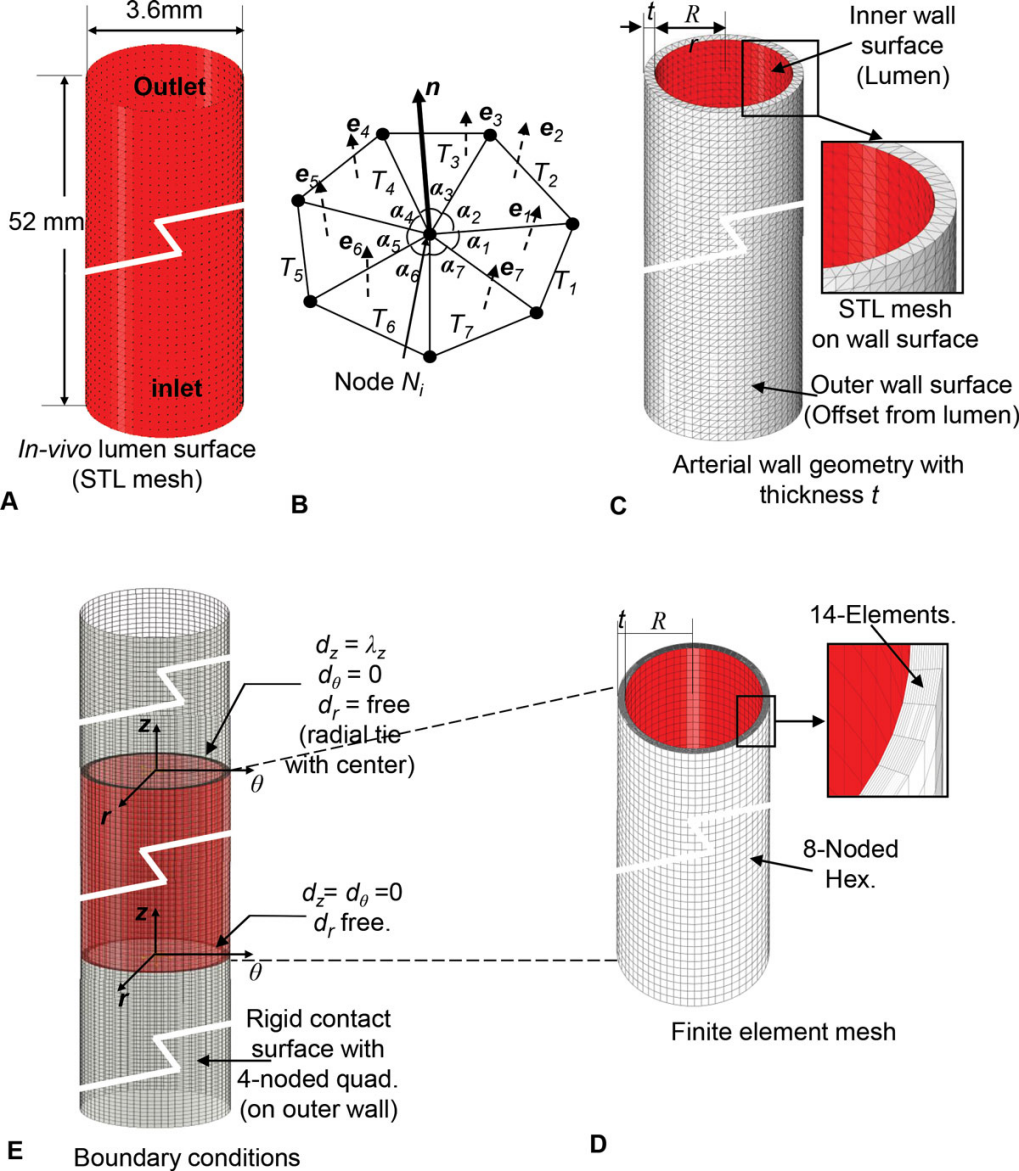
**Application of the inverse algorithm to a straight artery**. A) *In-vivo *lumen surface of STL triangles for idealized geometry of dog femoral artery (Sinha-Roy et al., 2008). B) Computation of nodal normal for offsetting lumen surface. C) Arterial wall geometry in form of STL mesh. D) Arterial wall mesh with 8 node hexahedral element. E) Finite element model with boundary conditions.

The lumen geometry is shown in Figure 1A, which can be obtained using image reconstruction if a patient-specific case is used. The outer wall surface of the artery was obtained by offsetting the lumen surface by computing an angle weighted normal at each node of the stitched STL triangles (Figure 1B). The outward normal direction was computed by,

(1) $$N=\frac{\underset{k}{\sum }\alpha_{k}e_{k}}{\underset{k}{\sum }\alpha_{k}},$$

where, ***e_1_***, ***e_2_***, ... are the unit normals of the ***k ***individual triangles, *T*_1_, *T*_2_, ... at a node of the STL mesh. The angles ***α_1_***, ***α_2_***, ... are the included angle for these triangles (Figure 1B). The position vector, ***R***, of a node on the outer wall surface was calculated from the corresponding position, ***r***, of the node on the inner wall by:

(2) $$R=r-t\hat{n}.$$

where, ***t ***is the *in-vivo *wall thickness and $\hat{n}=N/)∥N∥$ is the unit normal vector at the node (Figure 1B). The resulting wall geometry was obtained as a mesh of surface triangles as shown in Figure 1C. The wall geometry was meshed with 4-noded linear hexahedral elements with 15 elements across thickness (Figure 1D). This meshed geometry was used to construct the finite element model for the inverse algorithm described below (Figure 1E).

### Arterial wall material property

**Figure 2:**
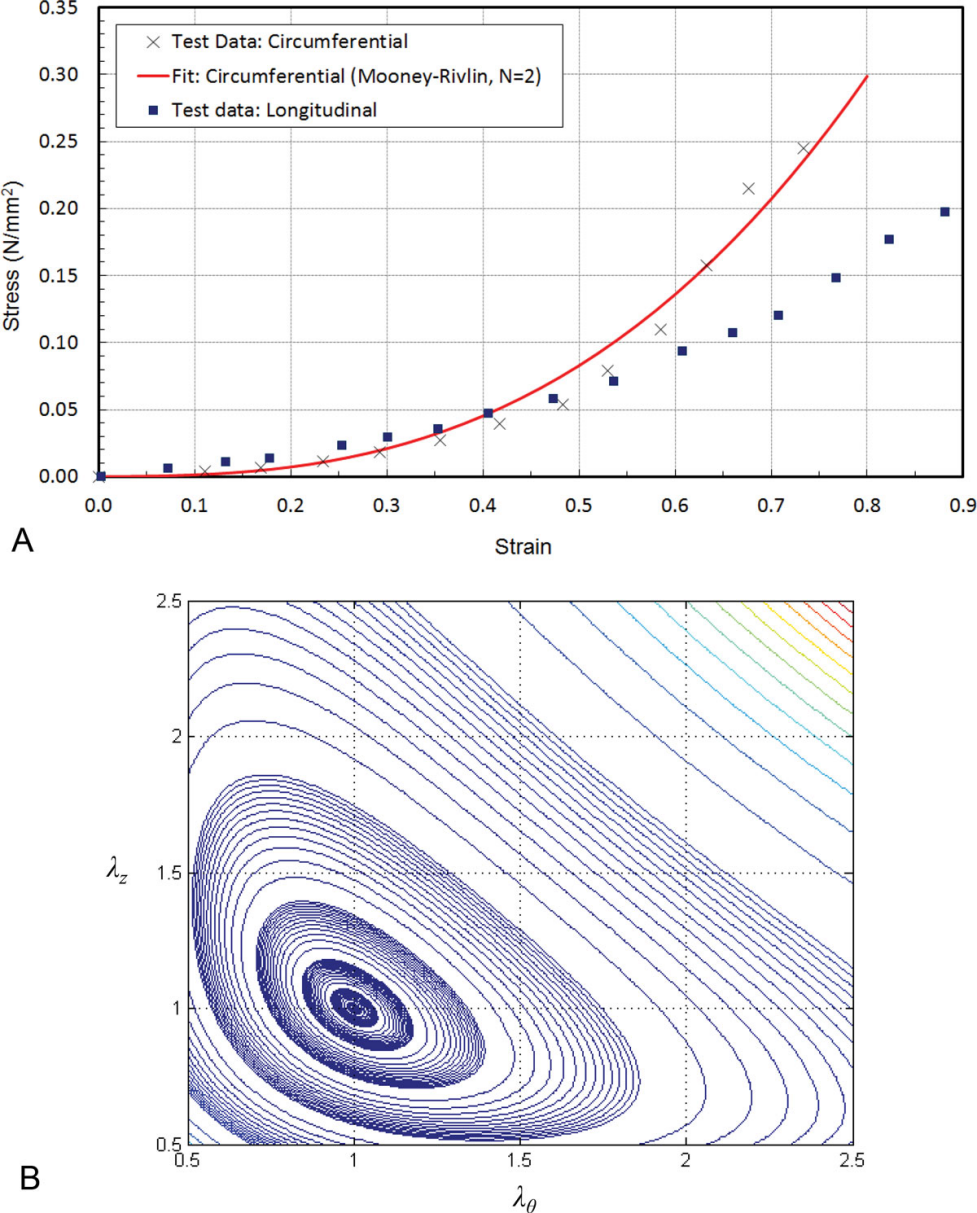
**Material model for arterial wall**. (A) Experimental data and curve-fit of the circumferential Cauchy stress and stretch data of dog femoral artery from Attinger et al., 1968. The curve-fit generated using generalized Mooney-Rivlin model with *N *= 2. (B) Contour plot of the corresponding strain energy density function.

The arterial wall material was modeled as homogeneous, incompressible, isotropic material of hyperelastic type. The constitutive equations for the wall material were derived using the generalized Mooney-Rivlin strain energy density function, W, of order *N *= 2

(3) $$W=\overset{N}{\underset{\begin{array}{c} p,q=0\\ p+q=1 \end{array}}{\sum }}C_{pq}{\left(I_{1}-3\right)}^{p}{\left(I_{2}-3\right)}^{q}$$

with invariants *I*_1 _and *I*_2 _[1,2,26,27]. The material constants: *C_10 _*= 1.157 × 10^-3 ^N/mm^2^, *C_01 _*= -0.314 × 10^-3 ^N/mm^2^, *C_20 _*= 13.689 × 10^-3 ^N/mm^2^, *C_11 _*= 7.942 × 10^-3 ^N/mm^2^, and *C_02 _*= 4.433 × 10^-3 ^N/mm^2 ^were obtained by nonlinear least square curve-fitting using the circumferential stress-strain data for the canine femoral artery obtained by Attinger, 1968 [28]. The plot of computed Cauchy stresses verses stretch in the circumferential direction is shown in Figure 2A along with the experimental data. Since the material model is isotropic, the longitudinal stress versus strain plot for the material will be same as the circumferential plot (Fit:Circumferential, Figure 2A). The experimentally obtained longitudinal stress versus stretch data is also plotted in the same figure for comparison. The material testing by Attinger (1968) was performed with an excised tubular-shaped sample of the artery [28]. Therefore, the constitutive model (Eq. 3), obtained by model-fitting the test data can be directly adopted for the cylindrical arterial geometry used in this study. It may also be noted that the cylindrical arterial specimen has residual stresses. Therefore, the residual stresses are indirectly included through the material model.

The contour plot of the strain energy density function, *W *(Eq. 3) with axial and circumferential stretch ratios, *λ_z _*and *λ_θ_*, show that the contour shapes are convex (Figure 2B). This shows that the strain energy density function, *W*, is positive definite. Moreover, the magnitude of strain energy density, *W*, at *λ_z _*= *λ_θ _*= 1 is 0.0 (Figure 2B). This demonstrates the validity of the material model obtained by curve-fitting.

### Inverse algorithm: load-free and pre-stressed wall geometry

**Figure 3:**
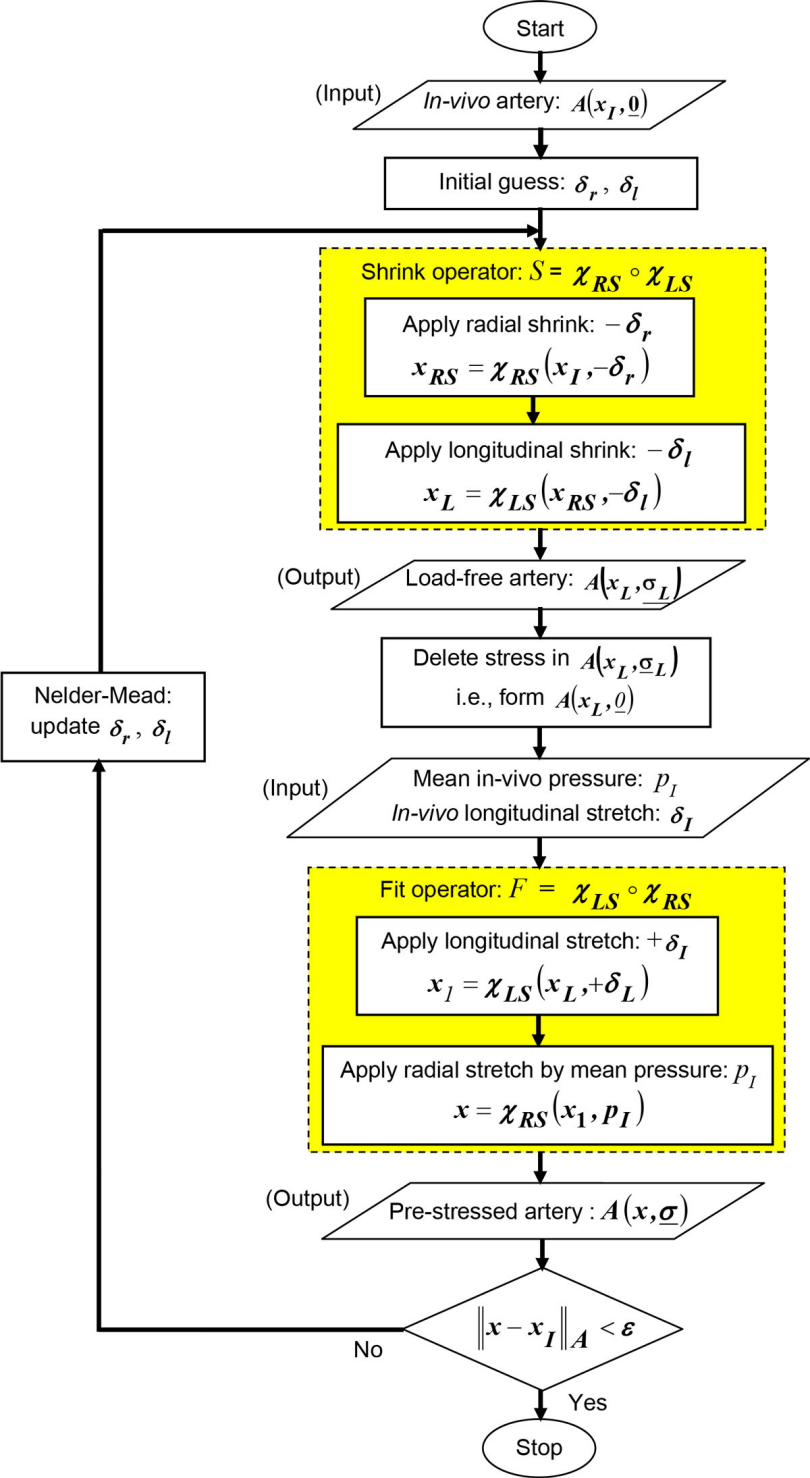
**Flow-chart of the inverse algorithm**. Block diagram of the *inverse *algorithm to compute the load-free and pre-stressed geometry. For *S *and *F*, ∘ denotes the composition of two operators, *f_1 _*and *f_2_*; defined as *f_2_*∘ *f_1 _*= *f_1_*(*f_2_*(*x*)).

The optimization-based inverse algorithm (Figure 3) was implemented using two operators: a) Shrink (*S*), and b) Fit (*F*). The operators, *S *and *F*, were based on two basic modes of wall deformation: radial deformation, ***χ_RS _***and longitudinal deformation, ***χ_LS_***, as described below. The shrink operator, *S*, shrinks the arterial geometry in the radial direction by ***δ_r_***, with the deformation operator ***χ_RS_***, followed by a longitudinal shrinking by ***δ_l_***, using the deformation operator ***χ_LS_***. The fit operator, *F*, was implemented to apply the *in-vivo *longitudinal stretch, ***δ_I _***and then apply mean arterial pressure, *p_I _*(Figure 3).

#### Longitudinal deformations, ***χ*_*LS*_**

The longitudinal deformation operator, ***χ_LS_***, was developed to perform the longitudinal shrinking or stretching of the artery while maintaining its *in-vivo *shape. It is denoted by ***χ_LS _***(***X***, ***δ_l_***), where ***X ***represents the arterial geometry it operates on and the real number, ***δ_l _***represents the incremental longitudinal deformation applied at the outlets of the arterial wall in the direction *d_z _*for extending or shrinking the wall geometry (Figure 1E). To compute the longitudinally shrunk (or stretched) arterial configuration, the operator performs a finite element solve with a frictionless, non-separating sliding contact surface that is coincident with the outer surface of the arterial wall to maintain the *in-vivo *arterial shape (Figure 1E). For a solution with ***δ_l _***> 0, ***χ_LS _***stretches the artery, whereas a solution with ***δ_l _***< 0 shrinks the artery radially. Since the sliding contact computation projects the arterial nodes on the rigid contact surface, this surface was extended at the inlet, as well as the outlet (Figure 1E), for ensuring a robust convergence of the contact solve.

#### Radial deformations, ***χ*_*RS*_**

The radial deformation operator performs radial expansion (stretch) or contraction (shrink) of the artery. The radial deformation can be performed in two ways: 1) a geometrical scaling by offsetting the nodes in the normal direction, $\hat{n}$ (Eq. 2) or 2) by expanding the arterial wall by applying a mean arterial pressure, *p_I_*. No wall stresses are produced in the process of radial deformation by geometrical scaling. However, stresses are generated in the process of radial expansion under the pressure load, *p_I_*. In a manner similar to ***χ_LS_***, the radial deformation is denoted by: ***χ_RS_*(*X*, *a*) **and its inputs are the arterial configuration represented by ***X ***and a real number ***a***. The parameter ***a ***can be pressure (*p_I_*) in the case of pressure induced deformation, or it can be a real number ***δ_r _***for the geometric scaling operation. The pressure induced radial deformation can only result in a radial expansion of the arterial wall. Applying a negative value of pressure at the inner wall is not feasible as it will lead to material instability. For geometric deformation by scaling, ***χ_RS _***can expand the artery radially by applying a radial stretch, ***δ_r_***, ***δ_r _***> 0, or it can shrink the artery radially by applying, ***δ_r _***< 0.

#### Inlet and outlet constraints

At each inlet and outlet cross-section, a local cylindrical coordinate system, (*d_r_*, *d_θ_*, *d_z_*), was created with its origin at the center of the cross-section. The coordinate system axis, *d_z _*was defined normal to the cross-sectional plane (Figure 1E). The nodal constraints for the nodes on the cross-section plane were defined with respect to this local cylindrical coordinate system. A reference node was created at the origin of the coordinate system and was held fixed to prevent any rigid body motion (Figure 1E). To allow the radial wall deformation due to pulsatile pressure, all nodes on the inlet and outlet were allowed to move in the radial direction from the reference node. The displacement of those nodes in the *d_z _*direction was prevented only at the inlet. At the outlets, motion of the nodes in the *d_z _*direction was allowed in order to apply longitudinal shrink or stretch.

#### Inverse algorithm

The inputs for the inverse algorithm are: 1) the unstressed *in-vivo *shape of the artery, 2) the mean *in-vivo *arterial pressure, *p_I _*, and 3) the *in-vivo *axial stretch, ***δ_I_***. For the femoral artery, *p_I _*was 104.1 mmHg and ***δ_I _***was considered to be 48% of the load-free artery length [4]. The inverse algorithm presented here, modifies the un-stressed *in-vivo *arterial geometry by performing the shrink and fit operations, resulting in new arterial configurations. To describe these configurations and their respective stress-states, a notation of ***A***(***x***,**σ**) has been used in which, ***x ***is used to denote a particular configuration, e.g., ***x_I _***for *in-vivo *and ***x_L _***for load-free, etc.; and **σ **is used to represent a 3x3 stress tensor for symbolically denoting the non-zero stress-state of the configuration. Therefore, ***A***(***x_I_***,***0***) is used to denote the un-stressed *in-vivo *arterial configuration, where ***0 ***represents the 3 × 3 null tensor. The steps (Figure 3) of the inverse algorithm are as follows:

1. Assume initial value of radial shrink, ***δ_r_***, and longitudinal shrink, ***δ_l_***.

2. Apply shrink (*S*) to the unstressed *in-vivo *artery geometry, ***A***(***x_I_***, ***0***). This is performed in two steps:

a. Apply radial shrink, ***χ_RS _***to the *in-vivo *geometry, *A*(***x_I_***, ***0***), to radially shrink by ***δ_r_***. That is: ***x_RS _***= ***χ_RS _***(***x_I_***, ***δ_r_***), where ***x_I _***is a point on the *in-vivo *artery geometry, and ***x_RS_***, its mapping on the radially shrunk artery.

b. Apply longitudinal shrink, ***χ_LS _***to the artery geometry resulting in the step 2a. That is: ***x_L _***= ***χ_LS_***(***x_RS_***, ***δ_l_***), where ***x_L _***is a point on the load-free artery after radial and longitudinal shrink. The resulting shrunk artery geometry, ***A***(***x_L_***,**σ_L_**) has stresses, ***σ_L_***.

3. Delete stresses, ***σ_L _***from ***A(x_L_***, ***σ_L_***) to obtain ***A***(***x_L_***, **0**). This is a trial load-free geometry.

4. Apply fit (*F*) to ***A***(***x_L_***, **0**) obtained in step 3. This is also performed in two steps:

a. Apply longitudinal stretch, ***χ_LS _***to stretch the *load-free *geometry, ***A***(***x_L_***, **0**) by ***δ_I_***. That is: ***x***_1 _= ***χ_LS_***(***x_L_***, ***δ_I_***), where ***x_1 _***is the location of ***x_L _***after longitudinal stretch.

b. Apply radial expansion, ***χ_RS_***, by applying *in-vivo *mean pressure, ***p_I _***. That is: ***x ***= ***χ_RS_***(***x_1_***, ***p_I_***), where ***x ***is a point on the pre-stressed artery. The result is a trial *pre-stressed *geometry, ***A***(***x***, **σ**), with stresses, **σ**.

5. Evaluate the least-square error function, ***ε***:

(4) $$\varepsilon ={∥x-x_{I}∥}_{\Omega }=\sqrt{\underset{\Omega }{\sum }{∥x-x_{I}∥}^{2}},$$

which is defined as the sum of the deviation of the nodal position of the nodes in the set **Ω**, between their location on the pre-stressed artery and their corresponding location, ***x_I_***, in the *in-vivo *artery. For this study, **Ω **was taken as the set of nodes on the outer surface of the arterial wall.

6. Stop the algorithm, if the value of **ε **is less than a pre-determined limit, *L*;

else, if ***ε ***>*L*, update, ***δ_r_***, and ***δ_l _***using Nelder-Mead optimization algorithm and proceed to the Step-2. The value of *L *was assumed to be 0.5 in this study.

It may be noted in Step-4, described above, that the pre-stressed arterial geometry was computed as a part of the inverse algorithm. Therefore, the two sequential outputs of the converged algorithm are: a) a load-free arterial geometry (Step 3); and b) the corresponding pre-stressed geometry (Step 4).

Any geometrical shape matching-based inverse algorithm has two key operators: 1) an *optimization operator *(Step-6) to compute a trial load-free geometry and 2) a *computational mechanics operator *(Step-2 and Step-4), to compute the deformed shape after the application of the *in-vivo *pressure and longitudinal stretch. It is well established that arterial wall materials are incompressible in nature. In the *computational mechanics operator*, this incompressible behavior is incorporated through the material model. In the present study, this has been achieved by using incompressible hyperelastic Mooney-Rivlyn material. However, for a realistic simulation, the *optimization operator *which computes a trial load-free arterial shape, also needs to incorporate this incompressibility constraint. In this research, this was done through an additional volumetric constraint to preserve the *in-vivo *arterial volume during optimization iteration.

#### Implementation and convergence

**Figure 4:**
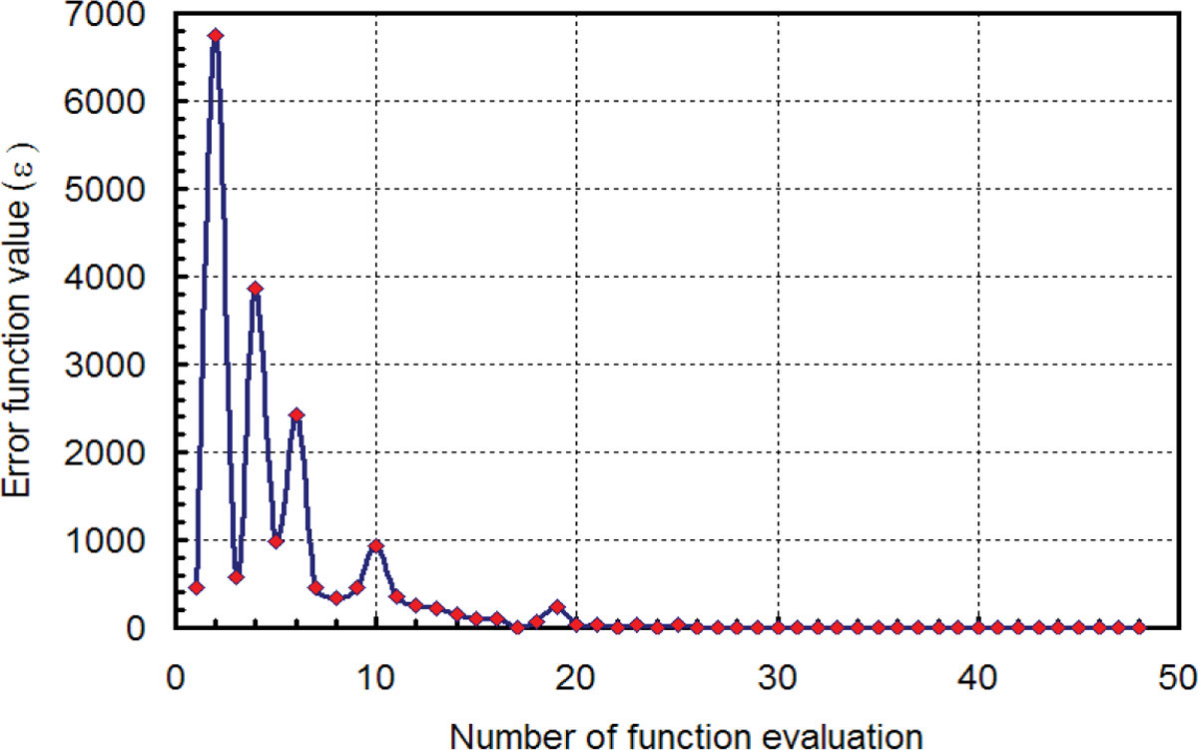
**Algorithm convergence**. Convergence of the inverse algorithm in terms of the objective function value and the number of function evaluation.

The complete methodology presented above was implemented using the python scripting language available in the ABAQUS CAE application (Dassault-Systems, Paris, France). The rate of convergence in terms of the value of the least-square objective function at the *i*-th evaluation from the beginning of the inverse process is shown in Figure 4. The *x*-axis shows the number of objective function evaluations from the start of the process. For the arterial geometry studied in this research, the algorithm was found to converge within 20 to 30 evaluations of the objective function. The pre-determined termination criteria, *L *= 0.5 or less was found to provide an acceptable optimal solution. In practice, the objective function values from the first few function evaluations will provide a reasonable initial guess for ***δ_l _***and ***δ_r_***.

### Pulsatile pressure-flow response

The equations of motion for the arterial wall and blood-flow along with the boundary conditions are described in the previously published research by Konala et al., [29]. The pulsatile pressure-flow response of the arterial wall was computed by solving the coupled equations of wall deformation and the hemodynamic equations of blood flow using ADINA (ADINA R & D, Inc., Watertown, MA). The non-Newtonian blood was modeled as a Carreau fluid [30].

**Figure 5:**
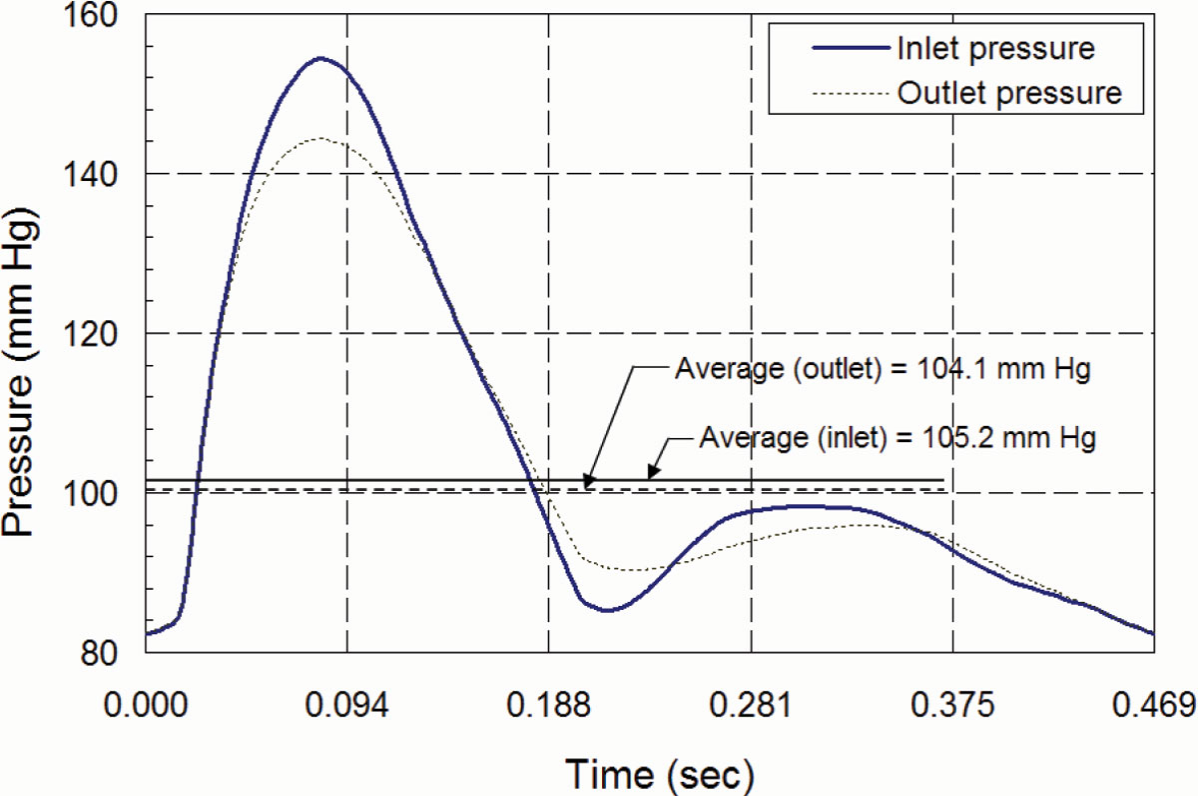
**Pulsatile boundary conditions**. Pulsatile pressure boundary condition *p_in_*(*t*) at the inlet, and *p_out_*(*t*) at the outlet, applied as normal traction for blood flow-wall interaction.

The dimensions of the load-free arterial geometry were obtained by the inverse method described above. An axisymmetric finite element model of the fluid and the structure was used for the pulsatile pressure-flow analysis [29]. The analysis was performed in two steps. In the first step, the load-free artery geometry, which was obtained in the Step 3 of the inverse algorithm, was pre-stressed by applying *in-vivo *longitudinal stretched, ***δ_I_***, and the mean *in-vivo *arterial pressure, *p_I_*. In the second step, the pulsatile pressure was applied to the pre-stressed artery. The pressure pulses, *p_in_*(*t*) and *p_out_*(*t*) were applied at the inlet and outlet, respectively (Figure 5), as normal surface tractions [13].

## Results

The results are presented in three sections. First, the results for dimensions of the load-free and pre-stressed geometry computed by the inverse algorithm are presented. Then the stresses and strains in the pre-stressed arterial wall are presented. Finally, the arterial wall stresses under the combined pulsatile pressure load and arterial wall pre-stress are presented.

### Load-free and pre-stressed arterial geometry

**Figure 6:**
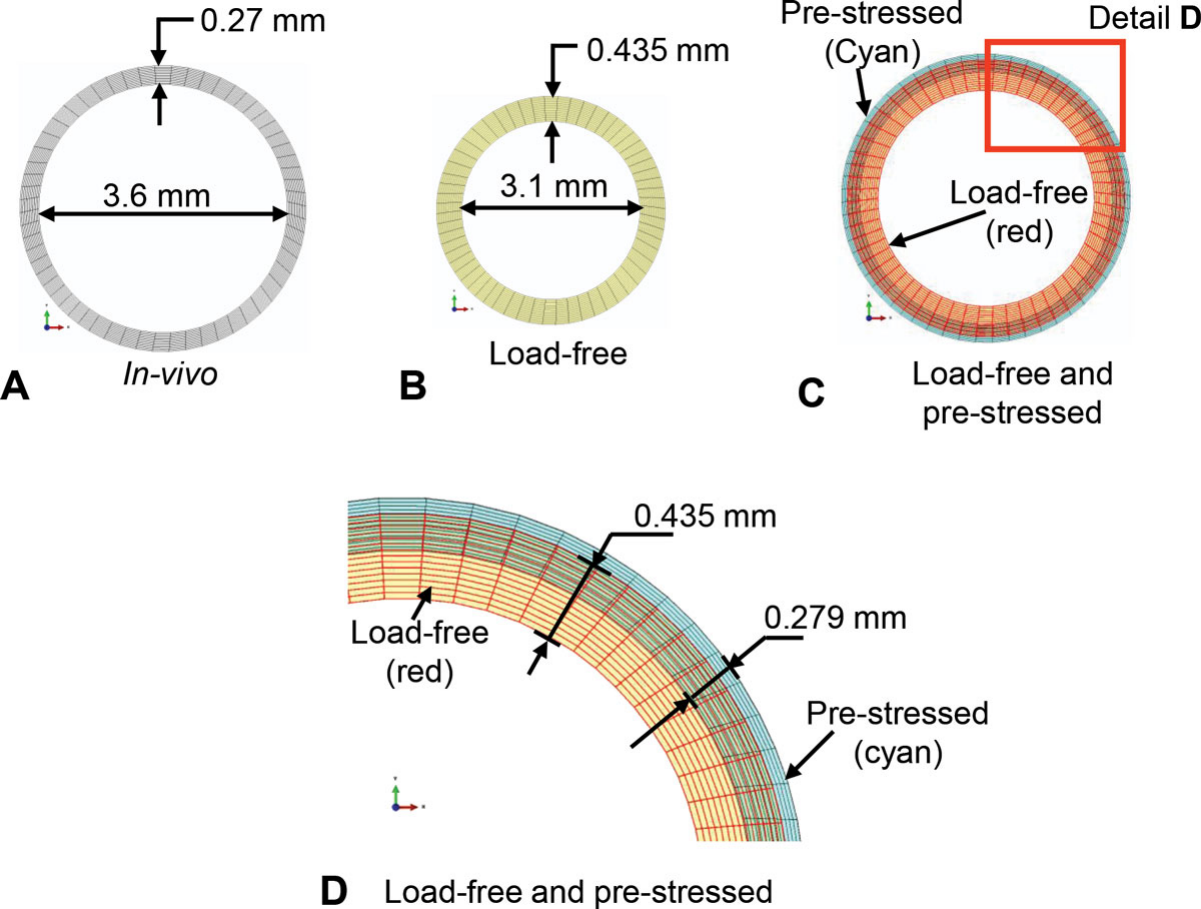
**Inverse algorithm results**. Results from the inverse algorithm to compute the load-free and the pre-stressed geometry for the idealized, straight, uniform diameter dog femoral artery model. Dimensions of: A) *in-vivo *wall; B) load-free wall; C) pre-stressed arterial wall with load-free wall superimposed on it. D) Details of the load-free and the pre-stressed cross-section. The comparison of the pre-stressed artery geometry with that of the *in-vivo *artery show that the two are within 0.0015 mm deviation of each other (the two nearly superimpose on one another).

The cross-sectional dimensions of the load-free and pre-stressed arterial geometry for the femoral artery using the inverse algorithm are presented in Figure 6. The *in-vivo *artery dimensions are shown in Figure 6A. The load-free artery dimensions computed by the inverse algorithm are shown in Figure 6B. The dimensions of the pre-stressed artery obtained by applying *in-vivo *longitudinal stretch and mean arterial pressure to the load-free geometry are shown in Figure 6C and 6D. For comparison, an image of the load-free geometry is also superimposed on the image of the pre-stressed artery.

The dimensions of the *in-vivo*, load-free and the pre-stressed artery are compared in Table 1. The length, inner diameter and thickness of the load-free arterial geometry calculated by the shrink-and-fit inverse algorithm are: 35.14 mm, 3.1 mm and 0.435 mm, respectively. The load-free artery length of 35.14 mm represents shrinkage of 32.4% from the *in-vivo *length of 52 mm. It also represents an axial stretch of 48% from the load-free length to the *in-vivo *length. The diameter change from the inner arterial wall, between the load-free artery (3.1 mm) and the *in-vivo *artery (3.6 mm) was 16.2%, whereas that for the outer arterial wall was 4.3%. The thickness of the load-free artery was 0.435 mm, which was 61% thicker than the *in-vivo *artery with thickness 0.27 mm.

**Table 1:** Inverse algorithm results.

		*In-vivo* (I)	Load-free (inverse)	Pre-stressed (P)	$\%=\left(\frac{I-P}{I}\right)\times 100$
Inner diameter (mm)	Inlet	3.60	3.104	3.604	1.1 × 10^-1^
	
	Outlet	3.60	3.104	3.604	1.1 × 10^-1^

Outer diameter (mm)	Inlet	4.14	3.974	4.140	0.0
	
	Outlet	4.14	3.974	4.140	0.0

Thickness	(mm)	0.270	0.435	0.269	3.7 × 10^-1^

Length	(mm)	52.0	35.13	51.99	2.0 × 10^-2^

Volume	(mm^3^)	170.69	169.90	169.90	4.6 × 10^-1^

The dimensions of load-free and pre-stressed geometry calculated by the inverse algorithm.

The length, inner diameter and the thickness of the pre-stressed artery obtained by applying a mean arterial pressure and axial stretch of 48% to the load-free geometry were: 51.99 mm, 3.603 mm and 0.2685 mm, respectively. The pre-stressed arterial geometry shows a reasonable match with the *in-vivo *shape as is evident from the comparison of the corresponding dimensions shown in Table 1. The difference in length between the *in-vivo *artery and the pre-stressed artery was 0.01 mm (0.019%). The inner wall diameter of the pre-stressed artery was within 0.004 mm (0.11%) of the *in-vivo *inner wall diameter of 3.6 mm. The difference in wall thickness between the *in-vivo *artery and pre-stressed artery was 0.0015 mm (0.37%).

The volume of the load-free as well as the pre-stressed artery was 169.90 mm^3^. This volume was calculated from the tessellated arterial geometry (Figure 2B). It was within 0.79 mm^3 ^of the *in-vivo *volume of 170.69 mm^3 ^since the optimization based inverse algorithm has preserved the material volume of the artery.

### Stresses and strains in pre-stressed artery

The stress, strain and deformation results for the pre-stressed artery under the mean *in-vivo *pressure of 104.1 mmHg and *in-vivo *longitudinal stretch of 48% from the load-free length are presented in this section. The value of the stresses, strains and deformations at the inner and the outer wall are tabulated in Table 2.

**Table 2:** Arterial wall stresses on inner and outer wall surface.

	σ*_rr _*× 10^1^	σ_θθ _× 10^1^	σ*_zz _*× 10^1^	ε*_rr _*× 10^1^	ε*_θθ _*× 10^1^	ε*_zz _*× 10^1^	*d_r _*× 10^1^
***r ***= ***r_i _***(Inner wall)	-0.137	1.271	1.937	-5.376	1.473	3.903	2.49

***r ***= ***r***_*0 *_(Outer wall)	-0.0015	0.708	1.404	-4.356	0.426	3.929	0.82

Stresses, logarithmic strains and radial deformation on the inner and outer arterial wall of the pre-stressed artery. Units for deformations are in mm and stresses in N/mm^2^.

The radial stress (σ*_rr_*) varies from the value of 0.0136 N/mm^2^, at the inner wall, equal to the applied pressure, to 0.0 N/mm^2 ^on the outer wall surface (Table 2). The change in circumferential stress from the inner wall surface (0.127 N/mm^2^) to the outer wall surface (0.071 N/mm^2^) was 44%. Due to the nonlinear material property, the longitudinal stress also varied across the vessel thickness. The difference in the longitudinal stress between the inner (0.193 N/mm^2^) and the outer wall (0.140 N/mm^2^) was 27.8%. In the pre-stressed configuration, the circumferential stresses are lower in magnitude than the longitudinal stresses. At the inner wall, the circumferential stress (0.127 N/mm^2^) was 34% lower than the longitudinal stress (0.193 N/mm^2^). Similarly, at the outer wall, the circumferential stress (0.071 N/mm^2^) was 49% lower than the longitudinal stress (0.140 N/mm^2^).

### Pulsatile flow rate

**Figure 7:**
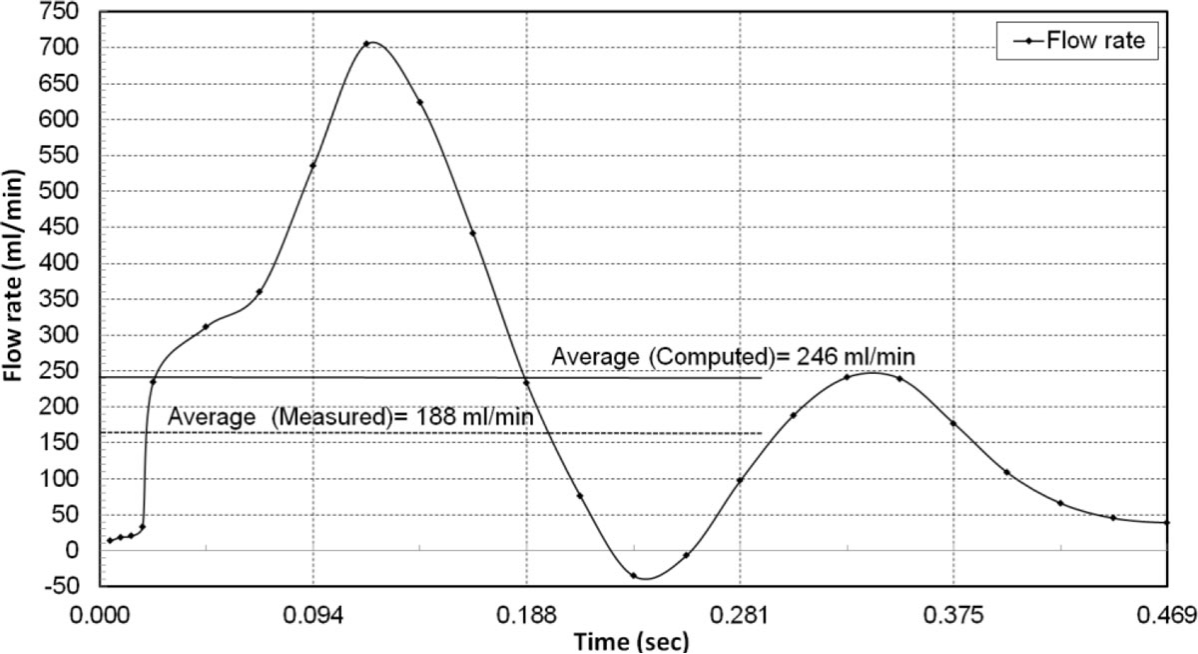
**Computed flow-rate under pulsatile pressure**. Transient flow rate under pulsatile pressure load with wall pre-stress.

The transient flow rate computed for the pre-stressed artery by applying the pulsatile pressure pulse is shown in Figure 7. The numerically computed time averaged flow rate, 246 ml/min, was 30% higher than the measured flow rate of 188 ml/min as reported by Sinha-Roy et al., [13], for the tapered femoral artery. The measured flow rate of 188 ml/min was obtained using Doppler flow wire for the tapered femoral artery. The difference between the computed and measured value could be because of Doppler measurements, which are based on average peak velocity (APV) and have been reported to register a lower flow rate than actual [31-33].

### Wall stresses under pulsatile pressure load

**Figure 8:**
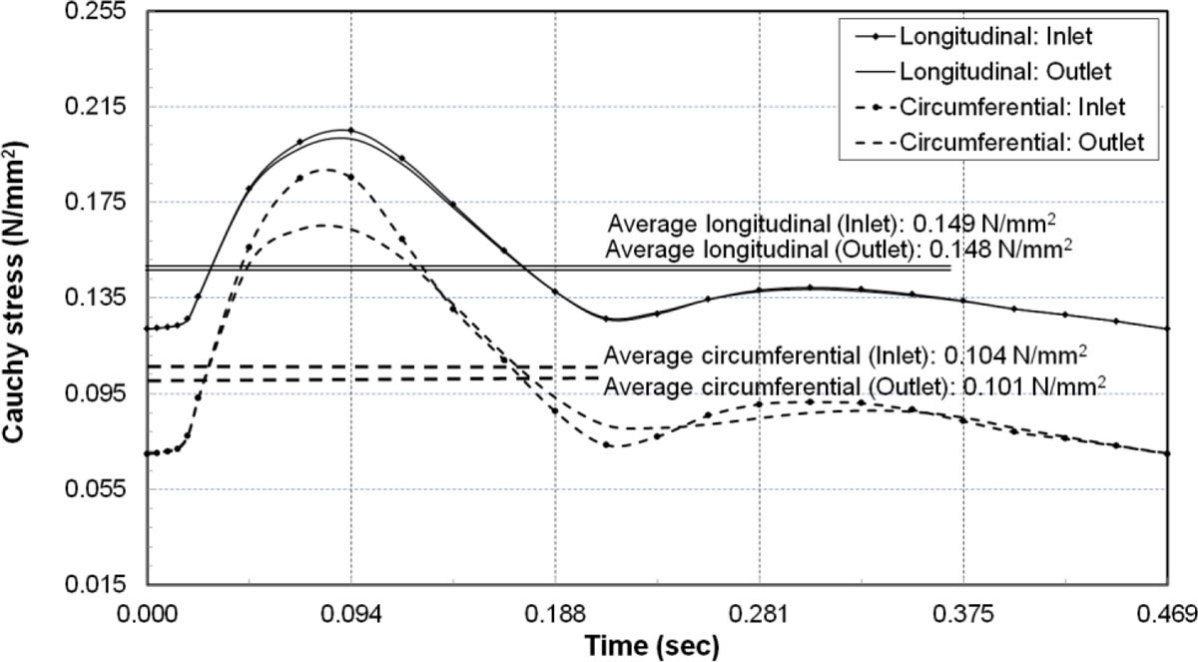
**Arterial wall stresses under pulsatile pressure load**. Cauchy stresses at the mid-wall location in the circumferential and longitudinal direction at the inlet and outlet.

The variation of the longitudinal and circumferential Cauchy stress over the cardiac cycle under the pulsatile inlet and outlet pressure pulse is presented in Figure 8. The time averaged value of Cauchy stress at the inlet in the longitudinal direction was 0.148 N/mm^2^, and that in the circumferential direction was 0.104 N/mm^2^. Therefore, for an *in-vivo *axial stretch of 48%, the time averaged longitudinal stress was 42.5% higher than the circumferential stress.

Due to the pulsatile flow, the time-averaged longitudinal stresses had a difference of 0.001 N/mm^2 ^between the inlet (0.149 N/mm^2^) and the outlet (0.148 N/mm^2^). Between the inlet (0.104 N/mm^2^) and the outlet (0.101 N/mm^2^), the corresponding difference in the circumferential stress was only 0.003 N/mm^2^. The peak stress in the circumferential direction was 0.187 N/mm^2 ^at the inlet and 0.168 N/mm^2 ^at the outlet, which corresponded to the time instant of peak pressure (Figure 5). Therefore, as evident from Figure 8, the overall time-variation of stress showed a similar trend between the inlet and outlet.

## Discussion

The primary contribution of this study was the development of an inverse algorithm to calculate the *in-vivo *arterial pre-stress for a patient-specific artery. This algorithm was developed for a patient-specific arterial geometry. For the present study, the algorithm was tested using an idealized arterial wall geometry that was cylindrical in shape. Even though this idealized geometry was axisymmetric, the algorithm was tested using a 3D cylindrical geometry. This was done to mimic the steps of a patient-specific case.

**Figure 9:**
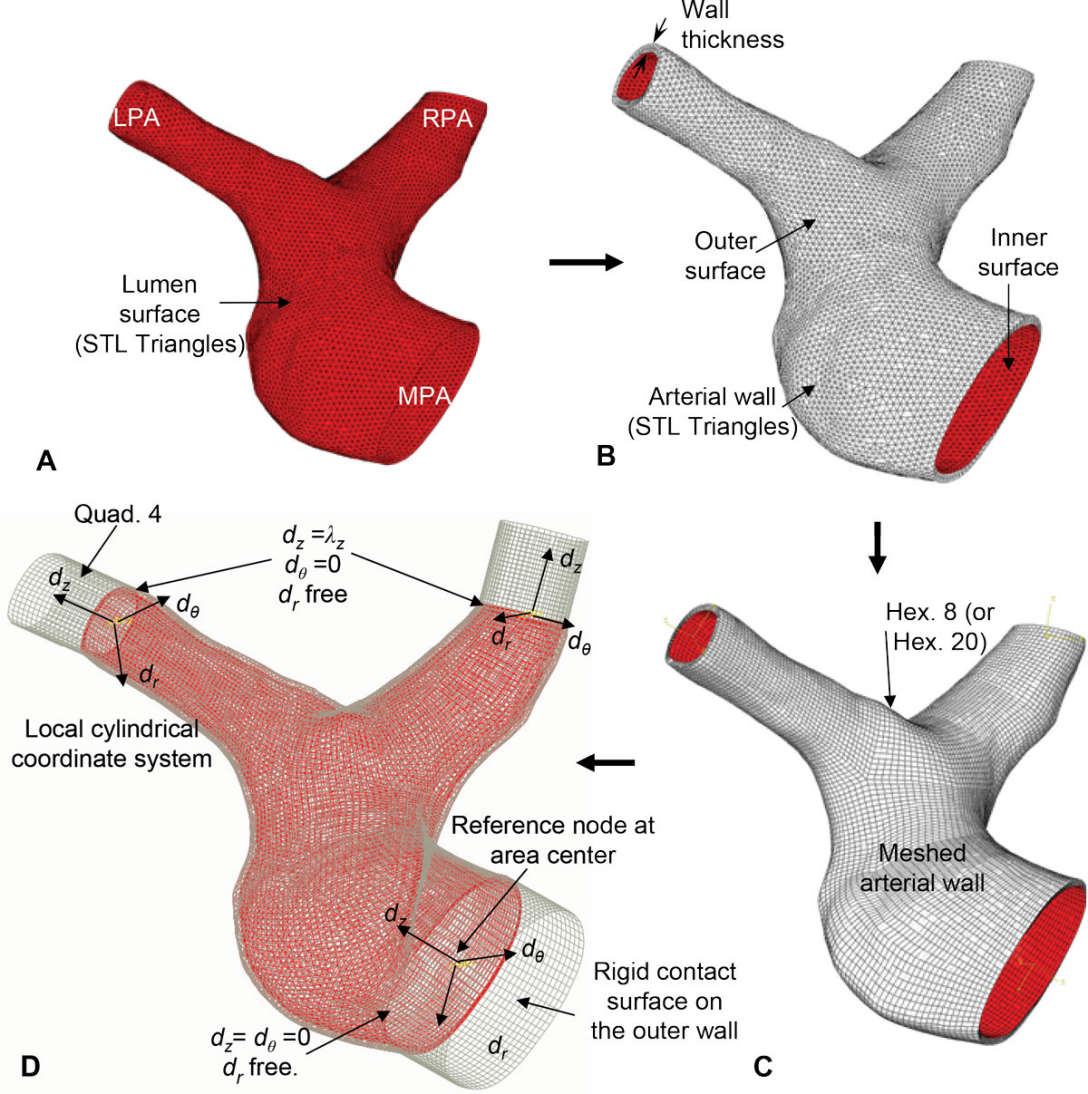
**Application of the inverse algorithm to a patient-specific artery**. Intermediate steps of the *inverse *algorithm. A) Lumen surface in form of triangular mesh of STL triangles obtained by geometry reconstruction. B) Arterial wall geometry in form of STL-mesh of surface triangles. C) Finite element mesh of the wall geometry using 8 node (or 20 node) hexahedral elements. D) Constraints imposed on the arterial wall motion in each shrink and fit iteration. Rigid contact surface superimposed on the outer arterial wall surface and extended at the ends, to maintain arterial shape during the longitudinal (axial) shrink or stretch operation. Contact surface meshed with 4-noded quadrilateral elements. Only in-plane radial motion in *d_r_*-*d_θ _*plane allowed for the nodes of the inlet and outlet surfaces. Additionally, nodes of the outlets are allowed to move in *d_z _*direction during longitudinal stretch or shrink operation.

In addition to the idealized axisymmetric geometry, the algorithm was tested for a 3D patient-specific arterial geometry. This was done to test the proposed steps (Figure 3) as described in the methods section, for a patient-specific case. The intermediate steps of the inverse algorithm for a patient-specific artery are shown in Figure 9. Specifically, Figure 9A shows the lumen boundary, which in the patient-specific case will be obtained from image reconstruction. As shown in Figure 1A, for the straight artery case, this surface is simply a cylindrical surface. Similarly, Figure 9B shows the arterial wall geometry obtained by adding wall thickness using nodal normal defined by Eq. 1. The corresponding wall geometry for the straight artery case (Figure 1C) was constructed by adapting the same procedure. Next, the finite element mesh used by the longitudinal and radial shrink operators for the patient-specific case is presented in Figure 9C, whereas Figure 1D shows the same for the straight artery. Finally, the boundary conditions and constraints imposed on the finite element mesh for the patient-specific case and the straight artery case are shown in Figure 9C and 1D, respectively. The complete pressure-flow analysis using blood-arterial wall interaction for such a patient-specific case will be presented in future.

The proposed algorithm simplified the inverse optimization problem to a two variable problem involving radial and axial deformation. This helped in substantially reducing the number of optimization variables from positions of each node to just two variables. Moreover, the incompressibility of arterial material was incorporated in the inverse optimization as a volumetric constraint; which preserved the *in-vivo *arterial volume during optimization iterations. The performance and robustness of the algorithm for patient-specific case needs to be tested with realistic artery geometry. For example, the algorithm employed a longitudinal wall deformation operator to shrink or stretch the arterial wall. The operator used sliding contact on a rigid contact surface, without contact separation, to deform the wall while not altering its *in-vivo *arterial shape. However, for a patient-specific case, the accuracy and performance of the contact algorithm is expected to be influenced by the complex and tortuous 3D wall shape.

In the implementation of the inverse method, the Nelder-Mead optimization algorithm was utilized for solving the non-linear least square minimization problem. One of the drawbacks of the Nelder-Mead algorithm is its slow convergence near the optimal solution. In addition to that, the convergence of Nelder-Mead to an optimal solution has not been proven mathematically. Other researchers have typically used the Levenberg-Marquardt (LM) algorithm for inverse computation. However, the advantage of Nelder-Mead is that it is a derivative free algorithm, whereas LM requires computation of derivatives. The computation of the derivatives can be expensive when a finite element solution is required for the evaluation of the objective function.

In the test with the idealized straight arterial geometry, the proposed inverse algorithm was found to converge to an acceptable solution within 20 to 25 evaluations of the objective function (Figure 4). However, as stated above, the convergence and robustness of the algorithm needs to be tested for a real patient-specific case.

The validity of the load-free geometry computed by the inverse algorithm was assessed by two criteria: 1) the geometrical match between the dimensions of the pre-stressed artery and the *in-vivo *artery, and 2) the change in diameter between the load-free artery and the pre-stressed artery. The first criterion is the necessary condition for a valid inverse solution. The second criterion is also important which is discussed here. It is possible to obtain a solution of the inverse problem satisfying the first criteria even if inaccurate arterial wall material properties are specified in the computational model. However, under such a scenario the deformation of the pre-stressed artery from the load-free configuration may not be accurate. For example, if the arterial wall material property is erroneously specified to be softer than what is actually observed, the deformation of the artery from the load-free to the pre-stressed configuration will be large. Excessive diameter changes between the calculated load-free geometry and the pre-stressed geometry will imply inaccurate load-free geometry. Ideally, the diameter change should match with the *in-vivo *measurements. For the femoral artery in the present study, the change in the outer diameter was 4.3% between the load-free (3.97 mm) and pre-stressed artery (4.14 mm). The corresponding change in the inner diameter was 14%. This was similar to the 5% outer diameter change reported by Huang et al., [5], for a human carotid artery using a direct method (based on trial and error procedure). Similarly, a change of 19% in the inner diameter of a porcine left anterior descending (LAD) artery was reported by Hamza et al., [7].

It may be noted that the pre-stresses in an arterial wall segment are the result of equilibrium of the wall segment (in the time averaged sense) under the applied stresses and stretch. This equilibrium is primarily a balance between the different stress components caused by the arterial pressure and tethering, along with the shear stresses due to the blood flow, in relation to the stretch ratios in the axial, circumferential and radial directions. Therefore, as indicated by Raghavan, et al., [34], some deviations in the arterial wall material property values do not significantly affect the results of the wall pre-stress. However, specification of significantly inaccurate material properties may result in an incorrect computation of the load-free arterial geometry.

The time-averaged flow rate computed with the pulsatile pressure was 30% higher than the measured value of 188 ml/min as reported by Sinha-Roy et al., [13]. The potential under-measurement than actual arterial flow rate by the Doppler flow wire, which measure flow rate based on APV could have contributed to this difference [31-33].

The magnitude of the average circumferential and longitudinal stress in the artery has been reported to depend on the value of the longitudinal stretch applied to an un-tethered and unloaded, *ex-vivo *artery. For porcine LAD, Zhang et al., [35], have reported lower magnitude of the average stress in the longitudinal direction compared to the circumferential direction when the axial stretch was less than 40%. They report that longitudinal stresses exceed circumferential stresses for axial stretch ratios greater than 1.4 (i.e., 40% stretch from load-free length). The present study shows a development of higher stresses in the longitudinal direction than circumferential, when the artery is subjected to an *in-vivo *longitudinal stretch of 48% from its load-free length. Specifically, under the pulsatile pressure, the mean stress in the longitudinal direction was 42.5% higher than the stress in the circumferential direction.

The factors involved in the computation of *in-vivo *arterial stress are: a) wall deformations, and b) wall material properties. The present results for the dog femoral artery can be affected by any of those factors. Zhang et al., [35] have reported that the use of an isotropic Mooney-Rivlin material model, instead of an anisotropic model, can result in a lower value of the computed circumferential stresses. Similarly, considerable variation in the *in-vivo *axial stretch along the arterial vasculature has been reported by many studies. These studies include, Guo et al., [36] for mouse aorta, Algranti et al., [8], for coronary arteries and Guo et al., [6], for coronary arteries, as well as veins. Guo et al., [6] study have also shown the dependence of *in-vivo *axial stretch on the vessel diameter. It is possible that the axial stretch of 48% for the diameter of the femoral artery evaluated in this study may need further investigation.

## Conclusions

In this study, a methodology has been developed to incorporate the arterial wall pre-stress in wall-blood flow interaction computation under pulsatile pressure. An optimization-based inverse algorithm has been developed and tested to incorporate arterial wall pre-stresses in the computational model. This algorithm was used to compute the load-free arterial geometry from the *in-vivo *arterial wall shape, axial stretch and mean arterial pressure. For the canine femoral artery, the resulting pre-stressed artery geometry, obtained by subjecting the computed load-free geometry to *in-vivo *pressure and longitudinal stretch, was within 0.0015 mm of the *in-vivo *geometry. The inverse algorithm has been designed to handle patient-specific cases. However, further testing is required for a patient-specific case with: a) the realistic material property, and b) an accurate measurement of the *in-vivo *longitudinal stretch.

Under pulsatile pressure and the *in-vivo *axial stretch of 48% from the load-free length, the arterial stress in the longitudinal direction was found to be 42.5% higher than those in the circumferential direction. This could be the result of either the relatively higher *in-vivo *stretch (48% of load-free length) considered in our computation or the use of an isotropic material model for the arterial wall material, rather than an anisotropic material formulation.

## Nomenclature

***x ***= a point of the pre-stressed artery.

***x_I _***= a point of the *in-vivo *artery.

***x_L _***= a point of the load-free artery.

***δ_r _***= radial deformation computed by optimization algorithm.

***δ_l _***= length of longitudinal or axial stretch (or shrinking) computed by the optimization algorithm.

***δ_I _***= *in-vivo *axial stretch.

*p_I _*= mean *in-vivo *pressure.

***χ_RS _***= radial deformation operator.

***χ_LS _***= longitudinal or axial deformation operator.

*S *= shrink operator to shrinks the arterial geometry in the radial and axial direction.

*F *= fit operator to deform artery by applying the *in-vivo *longitudinal stretch and mean *in-vivo *pressure.

**σ*_L _***= residual stresses in the load-free artery; a 3 × 3 tensor.

**σ **= stresses in pre-stressed artery; a 3 × 3 tensor.

***0 ***= zero stress state (at all points); a 3 × 3 null tensor.

***A***(***x_I_***, ***0***) = *in-vivo *arterial geometry without any stress.

***A***(***x_L_***, **σ*_L_***) = load-free arterial geometry with stresses, **σ*_L_***.

***A***(***x_L_***, **0**) = load-free arterial shape with stresses deleted.

***A***(***x***, **σ**) = pre-stressed artery with stresses, **σ**.

***ε ***= objective function for least-square minimization.

## Disclosure

The method presented in this manuscript is covered by a pending provisional patent (U.S. Serial No. 62/029028 dated July 25, 2014).

## Competing interests

The authors declare that they have no competing interests.

## Authors' contributions

AD did the study design, developed the inverse program, performed numerical calculations and analyzed results, and drafted the manuscript. AP contributed to technical discussion, meshing of the arterial wall geometry and reviewed the manuscript. RKB's contributions include study design, data interpretation, and approval of the final manuscript. MT was involved in technical discussion. All authors read and approved the final manuscript.

## Declarations

Publication charge for this article was paid with funding from Cincinnati Children's Hospital Medical Center (CCHMC).

This article has been published as part of *BioMedical Engineering OnLine *Volume 14 Supplement 1, 2015: Cardiovascular Disease and Vulnerable Plaque Biomechanics. The full contents of the supplement are available online at http://www.biomedical-engineering-online.com/supplements/14/S1
